# Neuronal Dysregulation in Stroke-Associated Pseudobulbar Affect (PBA): Diagnostic Scales and Current Treatment Options

**DOI:** 10.4172/2155-9562.1000323

**Published:** 2015-10-31

**Authors:** Paul A Lapchak

**Affiliations:** Department of Neurology & Neurosurgery, Cedars-Sinai Medical Center, Los Angeles, USA

**Keywords:** PBA, Dysregulation, Treatment, Depression, Laughing, Brainstem, Cerebellum, Mania, Robotripping, Antidepressants

## Abstract

Until recently there was little understanding of the exact pathophysiology and treatment choices for stroke patients with Pseudobulbar affect (PBA). PBA is typically characterized by outbursts or uncontrollable laughing or crying and in the majority of patients, the outbursts being involuntary and incompatible with the patients’ emotional state. PBA is a behavioral syndrome reported to be displayed in 28–52% of stroke patients with first or multiple strokes, and incidence may be higher in patients who have had prior stroke events, and higher in females. There is typically involvement of glutaminergic, serotoninergic and dopaminergic neuronal circuits of the corticolimbic-subcorticothalamic-pontocerebellar network. PBA is now understood to be a disinhibition syndrome in which specific pathways involving serotonin and glutamate are disrupted or modulated causing reduced cortical inhibition of a cerebellar/brainstem-situated “emotional” laughing or crying focal center. Stroke-induced disruption of one or more neuronal pathway circuits may “disinhibit” voluntary laughing and crying making the process involuntary. With a “new” treatment currently being marketed to treat PBA patients, this article will delve into the neurological and physiological basis for PBA in stroke, and review progress with the diagnosis and treatment of PBA.

## Pseudobulbar Affect (PBA) Incidence

Unpredictable and highly exaggerated episodes of crying or laughing that are dramatically incongruent with the context of a stroke patient’s situation are now commonly known as pseudobulbar affect (PBA). PBA has been referred to as pathological laughing and crying (PLC), emotional liability, emotional dysregulation, involuntary emotional expression disorder, and even emotional incontinence (EI). PBA is an emotional disturbance that occurs in patients secondary to a stroke or multiple strokes. In many patients, the episodes cannot be easily controlled voluntarily. Key characteristics of PBA are that episodes can last from seconds to many minutes, episodes are not stimulated by a specific situation, conversely, PBA occurs in situations making crying or laughing awkward, causing the patient to be agitated and embarrassed.

Statistics for PBA vary somewhat between source and depending on the year of the publication [1], but there appears to be an increase in prevalence due to the increased diagnosis of the condition. In the PBA Registry Series (i.e., PRISM) [1], Brooks et al. estimated that up to 2 million people have PBA based upon screening using an online survey; the results were questionable because it was deemed self-diagnosis and results were not independently confirmed. Moreover, there was no other disease condition associated with PBA diagnosis. A survey of literature from 1993-present [2–6] indicates that up to 52% of stroke patients may have PBA and that a greater percentage of women report and/or have PBA compared to men, suggesting a significant gender difference. In the analysis by Colamonico et al. [7], House et al. [8], and Kim [9], 11–34% of stroke patients were afflicted with PBA based upon results from various diagnostic scales. There may be a correlation with depressive state of the stroke patient and the incidence of PBA [8,10]. Therefore, a significant population of stroke patients needs a treatment for PBA.

## PBA Diagnosis

Currently, there are at least 5 useful and different rating scales performed either by the patient or the caregiver to “diagnose” the condition PBA, some of which are useful for differential diagnosis. The most commonly used scales are described below and presented in (Tables 1–4):

1. Center for Neurologic Study lability scale (CNS-LS), a validated scale [11] with score 7 (no PBA symptoms) to 35 (maximum score) [Moore et al [11]] Example Form 1 (Table 1).
2. 36-Item Short Form Health Survey (SF-36), a 36 question form that primarily rates quality of life and basic health inclusive of mental well-being with score 0, worst health to 100, best health [7,12,13] Example Form 2 (Table 2)
3. Center for Epidemiologic Studies depression 10-item short form (CES-D10), a 10 question form primarily targeting depressive behavior with a score of 10 or greater considered depressed. Note, there is a CES-D20 version of the form that is more comprehensive and a cutoff score of 16 is indicative of significant depressive symptomatology. Example CES-D10 Form 3 (7) (Table 3).
4. Work productivity and activity impairment (WPAI) questionnaire correlated work productivity (i.e.: assessing impairments in paid work and activities) with extent of impairment. Example Form 4 (7) (Table 4).
5. Visual analog scale quality of life quality of relationships (VAS QOL/QOR). The scale is particularly useful to determine the impact of PBA symptoms on QOL and QOR [7]; the higher the score the greater the negative impact of PBA on quality of life measures in the patient.

The recent articles by Colamonico et al. [7] and Brooks et al. [1] present a comprehensive overview and comparison of responses received from questionnaire takers using some of scales described above. The most consistent diagnosis of stroke patients with PBA resulted from the CNS-LS questionnaire, with use of the CES-D10 for diagnosis of PBA instead of depression, and it is clear that higher CNS-LS scores will negatively impact VAS QOL scores (higher score).

## Neuroanatomical Basis for PBA

Few stroke patient studies have documented and correlated the incidence of PBA with neuronal damage in specific brain regions. There is literature pertaining to PBA involvement of primary neurotransmitters pathways including disruption of glutamate and serotonin transmission: serotonin in the corticolimbic and/or cerebellar pathways may be involved in PBA, whereas widespread modulation of glutamate transmission would have an impact on PBA incidence.

This section will summarize known neuroanatomical changes synthesized from neurodegenerative disease patients with PBA to form a thesis. For example, Kim [14] studied a population of 25 patients presenting with first strokes and found that post-stroke emotional incontinence was associated with infarcts in the dorsal globus pallidus, primarily of serotonergic origin. In addition, PBA has been correlated with lesions in the frontal lobes and pathways descending to the brain stem, basilar pontine nucleus and to the cerebellum [15,16]. It is thought that the cerebellum neurotransmission, in particular corticopontine-cerebellar circuits causes impaired cerebellar control of emotional responses. Ahmed and Simmons [17] have proposed that PBA is a disinhibition syndrome in which specific pathways involving serotonin and glutamate are disrupted. If there is reduced cortical inhibition of a brain stem situated “emotional” center related to laughing and crying, stroke-induced disruption of the pathway may “disinhibit” voluntary laughing and crying [15,16], making the process involuntary. Parvizi and colleagues [16] have termed loss of cortical-cerebellar input control of emotions “dysmetria” of emotional expression.

There may also be a sensory and motor component regulating emotions: the cerebellum acting as a gate controller over direct input from the motor cortex and frontal and temporal lobes. Thus, since many PBA patients have right frontal lobe lesions, and left frontal and temporal lesions [17], it is also possible that damage to frontotemporal-subcortical circuits may be involved in PBA. For example, the motor cortex circuit may be modulated by inhibitory input from the somatosensory cortex. A lesion in the cortex may reduce the inhibitory input resulting in disinhibition of the cerebellar-controlled “emotions”.

Brain mapping studies using diffusion-tensor magnetic resonance imaging (MRI/DTI) and electroencephalography (EEG) suggests that reduced serotonin and dopamine transmission and enhanced glutamate transmission are key components in the emotional dysregulation [18]. Thus, with this limited information, the hypothesized treatment for PBA would enhancing both serotonin and dopamine pathways and decreasing glutamate receptor stimulation.

## Treatment Options

Pharmacological intervention to reduce the number of PBA episodes and improve QOL can be directed at dopamine, serotonin and glutamate receptors, enzymes involved in removing neurotransmitters from synapses (Table 5). For PBA, a series of clinical trials with selective serotonin reuptake inhibitors (SSRIs) such as fluoxetine, sertraline, and paroxetine have proven to be beneficial to the patient as have tricyclic antidepressants (TCA, i.e., nortriptyline); the efficacy of SSRI’s and TCA’s is related to the serotonergic function enhancing component of each drug. In stroke patients, in a clinical trial setting, nortriptyline [Sensoval, Aventyl, Pamelor, Norpress, Allegron, Noritren and Nortrilen] [19], citalopram [Celexa, Cipramil] [20] and imipramine [Tofranil] [21] were found to be effective, but these drugs were never FDA-approved for the treatment of PBA. Moreover, there are sporadic reports of selective noradrenergic reuptake inhibitors (duloxetine [Cymbalta], venlafaxine and roboxetine) having some efficacy to treat PBA [22–24]. This long series of drug remain as possible choices off-label therapy choices for PBA patients who do not respond or fully respond to the current FDA-approved treatment (see below).

Glutamate antagonists, in particular the cough suppressant dextromethorphan [17,25,26], which can inhibit glutamate activity via the classical N-methyl-D-aspartate (NMDA) receptor as well as sigma 1 (σ1) receptors, but these activities are also related to toxicity (mania and robotripping) with excessive administration and overuse of the drug [26–32]. Drug metabolites also have potent activities at NMDA and σ receptors [25].

Surprisingly, the combination of dextromethorphan and quinidine (Nuedexta) is currently the only FDA-approved treatment for PBA. Nuedexta contains two different components; dextromethorphan hydrobromide to act on sigma-1 and NMDA receptors in the brain, and quinidine sulfate, a metabolic inhibitor (i.e.: a specific inhibitor of cytochrome P450 2D6 (CYP2D6)–dependent oxidative metabolism), and a class I antiarrhythmic agent [(by blocking the fast inward sodium current (Ina)] that enables dextromethorphan to reach therapeutic concentrations (see http://www.avanir.com/nuedexta). The proposed mechanism of action of the therapeutic does not seem to directly target dopamine and serotonin dysfunction in PDA, leaving new avenues open for PBA treatment using multi-drug combinations.

Clinical studies with Nuedexta in Multiple sclerosis patients demonstrate efficacy with a significant mean decrease of 7.7 to 8.2 vs 3.3 to 5.7 in placebo-treated patients using CNS-LS scores; the absolute benefit is 2.5–4.4 points as documented by Pioro et al. [33] and Panitch et al. [34]. In stroke patients, in PRISM II, a 7.7 point improvement in CNS-LS has been reported with a 75.5% reduction in PBA episodes compared to baseline [35].

## Conclusion

PBA is due to the dysregulation of 3 main neurotransmitter pathways, dopamine, serotonin and glutamate, from the frontal cortical lobes through the cerebellum and brain stem, the corticolimbic-subcorticothalamic-ponto-cerebellar network. Stroke or infarct-mediated interruption of circuits projecting to the cerebellum and brainstem may result in disinhibition of well-controlled voluntary emotions, making them involuntary. With Nuedexta being FDA-approved as a treatment for PBA, and with only modest, but statistically significant efficacy of the drug combination, new treatment options targeting dopamine and serotonin, in addition to glutamate, should be pursued to provide stroke patients the best opportunity to improve QOL by reducing PBA episodes.

## Figures and Tables

**Table 1:** CNS-LS questionnaire.

**Using the scale below, please write the number that describes the degree to which each item applies to you DURING THE PAST WEEK. Write only 1 number for each item.**				

**Never**	**Rarely**	**Occasionally**	**Frequently**	**Most of the time**
**1**	**2**	**3**	**4**	**5**
There are times when I feel fine 1 minute, and then I’ll become tearful the next over something small or for no reason at all. Others have told me that I seem to become amused very easily of that I seem to become amused about things that really aren’t funny. I find myself crying very easily I find that even when I try to control my laughter, I am often unable to do so. There are times when I won’t be thinking of anything happy or funny at all, but then I’ll suddenly be overcome by funny or happy thoughts. I find that even when I try to control my crying, I am often unable to do so. I find that I am easily overcome by laughter. **Score 7 (no PBA symptoms) to 35 (maximum score)**				

**Table 2:** SF-36 questionnaire.

**Please answer the 36 questions of the Health Survey completely, honestly, and without interruptions.**					
**CIRCLE YOUR BEST ANSWER**					
**1) GENERAL HEALTH: In general, would you say your health is:**					
Excellent	Very Good	Good	Fair	Poor	
**2) Compared to one year ago, how would you rate your health in general now?**					
Much better	Somewhat better	About the same	Somewhat worse	Much worse	
**Limitations of Activities: The following items are about activities you might do during a typical day.**					
**Does your health now limit you in these activities? If so, how much?**					
**3) Vigorous activities, such as running, lifting heavy objects, participating in strenuous sports.**					
Yes, Limited a lot	Yes, Limited a Little	No, Not Limited at all			
**4) Moderate activities, such as moving a table, pushing a vacuum cleaner, bowling, or playing golf**					
Yes, Limited a Lot	Yes, Limited a Little	No, Not Limited at all			
**5) Lifting or carrying groceries**					
Yes, Limited a Lot	Yes, Limited a Little	No, Not Limited at all			
**6) Climbing several flights of stairs**					
Yes, Limited a Lot	Yes, Limited a Little	No, Not Limited at all			
**7) Climbing one flight of stairs**					
Yes, Limited a Lot	Yes, Limited a Little	No, Not Limited at all			
**8) Bending, kneeling, or stooping**					
Yes, Limited a Lot	Yes, Limited a Little	No, Not Limited at all			
**9) Walking more than a mile**					
Yes, Limited a Lot	Yes, Limited a Little	No, Not Limited at all			
**10 Walking several blocks**					
Yes, Limited a Lot	Yes, Limited a Little	No, Not Limited at all			
**11) Walking one block**					
Yes, Limited a Lot	Yes, Limited a Little	No, Not Limited at all			
**12) Bathing or dressing yourself**					
Yes, Limited a Lot	Yes, Limited a Little	No, Not Limited at all			
**Physical Health Problems: During the past 4 weeks, have you had any of the following problems with your work or other regular daily activities as a result of your physical health?**					
**13) Cut down the amount of time you spent on work or other activities**					
Yes	No				
**14) Accomplished less than you would like**					
Yes	No				
**15) Were limited in the kind of work or other activities**					
Yes	No				
**16) Had difficulty performing the work or other activities (for example, it took extra effort)**					
Yes	No				
**Emotional Health Problems:**					
**During the past 4 weeks, have you had any of the following problems with your work or other regular daily activities as a result of any emotional problems (such as feeling depressed or anxious)?**					
**17) Cut down the amount of time you spent on work or other activities**					
Yes	No				
**18) Accomplished less than you would like**					
Yes	No				
**19) Didn’t do work or other activities as carefully as usual**					
Yes	No				
**20) SOCIAL ACTIVITIES: Emotional problems interfered with your normal social activities with family, friends, neighbors, or groups?**					
Not at all	Slightly	Moderately	Severe	Very Severe	
**21) PAIN: How much bodily pain have you had during the past 4 weeks?**					
None	Very Mild	Mild	Moderate	Severe	Very Severe
**22) During the past 4 weeks, how much did pain interfere with your normal work (including both work outside the home and housework)?**					
Not at all	A little bit	Moderately	Quite a bit	Extremely	
**Energy and Emotions:**					
**These questions are about how you feel and how things have been with you during the last 4 weeks. For each question, please give the answer that comes closest to the way you have been feeling.**					
**23) Did you feel full of pep?**					
All of the time	Most of the time	A good Bit of the Time	Some of the time	A little bit of the time	None of the Time
**24) Have you been a very nervous person?**					
All of the time	Most of the time	A good Bit of the Time	Some of the time	A little bit of the time	None of the Time
**25) Have you felt so down in the dumps that nothing could cheer you up?**					
All of the time	Most of the time	A good Bit of the Time	Some of the time	A little bit of the time	None of the Time
**26) Have you felt calm and peaceful?**					
All of the time	Most of the time	A good Bit of the Time	Some of the time	A little bit of the time	None of the Time
**27) Did you have a lot of energy?**					
All of the time	Most of the time	A good Bit of the Time	Some of the time	A little bit of the time	None of the Time
**28) Have you felt downhearted and blue?**					
All of the time	Most of the time	A good Bit of the Time	Some of the time	A little bit of the time	None of the Time
**29) Did you feel worn out?**					
All of the time	Most of the time	A good Bit of the Time	Some of the time	A little bit of the time	None of the Time
**30) Have you been a happy person?**					
All of the time	Most of the time	A good Bit of the Time	Some of the time	A little bit of the time	None of the Time
**31) Did you feel tired?**					
All of the time	Most of the time	A good Bit of the Time	Some of the time	A little bit of the time	None of the Time
**Social Activities:**					
**32) During the past 4 weeks, how much of the time has your physical health or emotional problems interfered with your social activities (like visiting with friends, relatives, etc.)?**					
All of the time	Most of the time	A good Bit of the Time	Some of the time	A little bit of the time	None of the Time
**General Health: How true or false is each of the following statements for you?**					
**33) I seem to get sick a little easier than other people**					
Definitely true	Mostly true	Don’t know	Mostly false	Definitely false	
**34) I am as healthy as anybody I know**					
Definitely true	Mostly true	Don’t know	Mostly false	Definitely false	
**35) I expect my health to get worse**					
Definitely true	Mostly true	Don’t know	Mostly false	Definitely false	
**36) My health is excellent**					
Definitely true	Mostly true	Don’t know	Mostly false	Definitely false	

**Table 3:** CES-D10 short.

Items:	Rarely or none of the time (less than 1 day)	Some or a little of the time (1–2 days)	Occasionally or a moderate amount of time (3–4 days)	All of the time (5–7 days)
**1. I was bothered by things that usually don’t bother me.**				
**2. I had trouble keeping my mind on what I was doing.**				
**3. I felt depressed.**				
**4. I felt that everything i did was an effort.**				
**5. I felt hopeful about the future.**				
**6. I felt fearful.**				
**7. My sleep was restless.**				
**8. I was happy.**				
**9. I felt lonely.**				
**10. I could not “get going”.**				

**Table 4:** WPAI questionnaire (Short-specific health problem).

The following questions ask about the effect of your PROBLEM on your ability to work and perform regular activities. *Please fill in the blanks or circle a number, as indicated.*
1. Are you currently employed (working for pay)? _____ NO ___ YES
*If NO, check “NO” and skip to question 6.*
The next questions are about the **past seven days**, not including today.
2. During the past seven days, how many hours did you miss from work because of problems associated with your PROBLEM? *Include hours you missed on sick days, times you went in late, left early, etc., because of your PROBLEM. Do not include time you missed to participate in this study. _____* HOURS
3. During the past seven days, how many hours did you miss from work because of any other reason, such as vacation, holidays, time off to participate in this study? _____HOURS
4. During the past seven days, how many hours did you actually work? _____HOURS *(If “0”, skip to question 6.)*
*5.* During the past seven days, how much did your PROBLEM affect your productivity while you were working?
Think about days you were limited in the amount or kind of work you could do, days you accomplished less than you would like, or days you could not do your work as carefully as usual. If PROBLEM affected your work only a little, choose a low number. Choose a high number if PROBLEM affected your work a great deal.
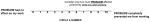
**6.** During the past seven days, how much did your PROBLEM affect your ability to do your regular daily activities, other than work at a job?
*By regular activities, we mean the usual activities you do, such as work around the house, shopping, childcare, exercising, studying, etc. Think about times you were limited in the amount or kind of activities you could do and times you accomplished less than you would like. If PROBLEM affected your activities only a little, choose a low number. Choose a high number if PROBLEM affected your activities a great deal.*
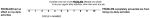

**Table 5:** Treatment of PBA.

Drug	Clinical Trial Design	Dose	Effect
Citaprolam [20] [Sensoval, Aventyl, Pamelor, Norpress, Allegron, Noritren, Nortrilen]	Double-blind, placebo-controlled, crossover	10–30 mg/day	Fifty percent decrease in crying episodes over a 9-week trial period.
Dextromethorphan/Quinidine [25,27,33,35] [Nuedexta]	Randomized, double-blind trial	20 mg/10 mg bid (USA and Europe); 30 mg/10 mg (Europe)	Significant mean decrease using CNS-LS of 7.7 to 8.2 vs 3.3 to 5.7 in placebo in MS patients; in stroke patients, a 7.7 point improvement in CNS-LS has been reported with a 75.5% reduction in PBA episode count compared to baseline.
Fluoxetine [36,37] [Sarafem]	Double-blind, placebo-controlled study	20 mg/day	Improvement in both emotional incontinence (PSEI), or anger proneness (PSAP), but not post-stroke depression (PSD).
Imipramine [21] [Tofranil]	Double-blind, placebo-controlled, crossover	10–20 mg/day	Overall improvement compared to placebo
Nortriptyline [19] [Celexa, Cipramil]	Randomized, double-blind, placebo-controlled	Dose escalation to 100 mg	Lowered pathological laughing and crying using PLACS scores.
Sertraline [38] [Zoloft]	Double-blind, randomized controlled trial	50 mg/day	Global health improvement and decreased crying.
